# Presence of Urinary Exosomes for Liquid Biopsy of Clear Cell Renal Cell Carcinoma: Protocol for a Pilot Feasibility Study

**DOI:** 10.2196/24423

**Published:** 2021-07-20

**Authors:** Guorong Li, Nora Mallouk, Pascale Flandrin, Arnauld Garcin, Claude Lambert, Sid Ali Berremila, Hocine Habchi, Nicolas Mottet

**Affiliations:** 1 Department of Urology North Hospital, CHU Saint-Etienne Saint-Etienne France; 2 Center of Electronic Microscopy Faculty of Medicine University Jean Monnet Saint-Etienne France; 3 Laboratory of Molecular Biology North Hospital, CHU Saint-Etienne Saint-Etienne France; 4 Unité de Recherche Clinique Innovation et Pharmacologie North Hospital, CHU Saint-Etienne Saint-Etienne France; 5 Laboratory of Immunology North Hospital, CHU Saint-Etienne Saint-Etienne France; 6 Laboratory of Pathology North Hospital, CHU Saint-Etienne Saint-Etienne France

**Keywords:** liquid biopsy, urine exosome, CA9, clear cell renal cell carcinoma, kidney cancer

## Abstract

**Background:**

Approximately 70%-80% of kidney cancers are clear cell renal cell carcinomas (CCRCCs). Patient management is based on imaging (abdominal ultrasound and computerized tomography), surgical excision of the tumor, and pathological analysis. A tissue biopsy is therefore necessary to confirm the diagnosis and avoid unnecessary nephrectomy. For metastatic cancers, a tissue biopsy is essential for establishing the targeted therapy. This biopsy of tumor material is invasive and painful. Other techniques such as liquid biopsy would help reduce the need for tissue biopsy. The development of a simple biological test for diagnosis is essential. CA9 is a powerful marker for the diagnosis of CCRCC. Exosomes have become a major source of liquid biopsy because they carry tumor proteins, RNA, and lipids. Urine is the most convenient biological liquid for exosome sampling.

**Objective:**

The aim of this study (PEP-C study) is mainly to determine whether it is possible to detect urinary exosomal CA9 for the molecular diagnosis of CCRCC.

**Methods:**

This study will include 60 patients with CCRCC and 40 noncancer patients. Exosomes will be isolated from urine samples and exosomal CA9 will be detected by transmission electron microscopy, flow cytometry, and reverse transcription-quantitative polymerase chain reaction.

**Results:**

This study is currently underway with funding support from the CHU Saint-Etienne of France.

**Conclusions:**

We expect to demonstrate that urinary tumor exosomes could be a novel liquid biopsy to diagnose CCRCC and to guide clinicians in treatment decision-making.

**Trial Registration:**

ClinicalTrials.gov NCT04053855; https://clinicaltrials.gov/ct2/show/NCT04053855

**International Registered Report Identifier (IRRID):**

DERR1-10.2196/24423

## Introduction

Renal cell carcinomas are serious and common cancers [1]. Approximately 70%-80% of kidney cancers are clear cell renal cell carcinomas (CCRCCs). Patient management is based on imaging (abdominal ultrasound and scanner), surgical excision of the tumor, and pathological analysis. Approximately one-third of patients with CCRCC have a locally advanced or metastatic tumor at the time of diagnosis and nearly 30% of treated patients will develop secondary metastases [1]. Recent advances in imaging techniques have increased the detection of kidney tumors; however, not all tumors are cancerous, with 10%-15% of all kidney tumors being benign and 20%-30% of tumors smaller than 4 cm being benign [2]. A tissue biopsy is therefore necessary to confirm the diagnosis and avoid unnecessary nephrectomy. For metastatic cancers, tissue biopsy is also essential for establishing the targeted therapy. This biopsy of tumor material is invasive and painful. Alternative techniques such as liquid biopsy would help reduce the rate of tissue biopsy. Therefore, development of a simple biological test for diagnosis is essential.

The notion of liquid biopsy has been mentioned in recent publications [3]. Liquid biopsies are based on the detection of cancerous biomarkers in body fluids, mostly in the blood, but can also include the urine, saliva, or other fluid. Liquid biopsies are preferable to tissue biopsies because they are less invasive.

Exosomes are small nanoparticles that are secreted into the extracellular medium by different cell types [4,5]. We propose detecting urinary exosomes from patients with CCRCC. A urine sample would be preferable to a blood sample because of the anatomical proximity of the urine to the kidney tumor. Moreover, urine is the simplest biofluid that can be obtained. We and others have found that urine is an important target for the exploration of markers for renal diseases [6]. The tumor exosomes from CCRCC in the urine are particularly abundant because of the location of the tumor directly in contact with the urine. Our hypothesis is that tumor exosomes present in the urine of patients with CCRCC could be detected. Our aim is to develop a reliable technique for the detection of tumor exosomes in the urine of patients with CCRCC to explore their usefulness as a liquid biopsy of CCRCC.

## Methods

### Study Design

**Figure 1:**
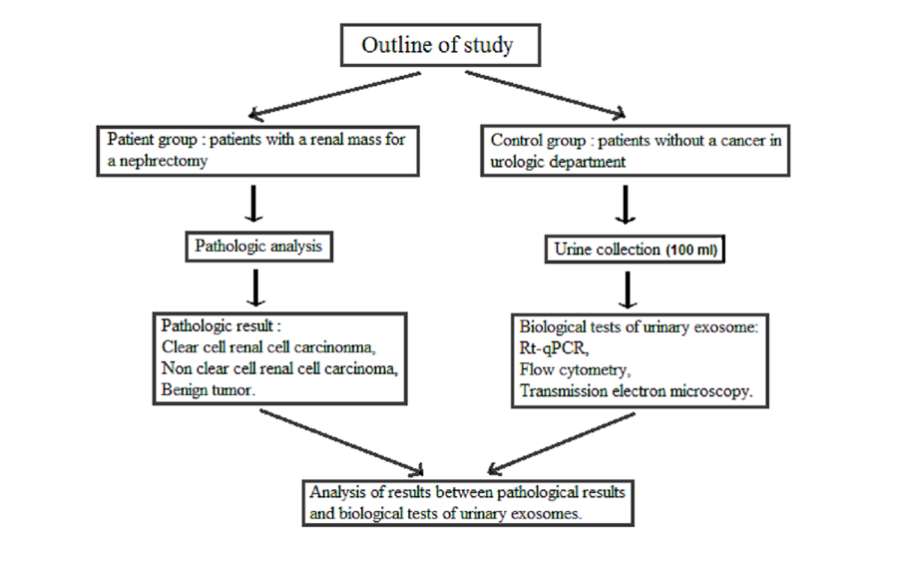
Outline of study. RT-qPCR: reverse transcription-quantitative polymerase chain reaction

This is a descriptive study (PEP-C study). The investigators will provide eligible patients with an informative notice on the PEP-C study. Upon a patient providing written consent, 100 ml of urine will be collected in a sterile tube for each exosomal analysis. The study design is outlined in Figure 1.

### Study Population

There is no available publication in the literature to calculate the number of subjects required for this type of study. Thirty patients receive surgery for removal of CCRCC in our department per year. Therefore, we propose to study 60 patients over 2 years. In addition, 20 control patients without cancer in our department are expected per year. Therefore, we propose studying 40 controls over 2 years. Eligibility criteria for cancer patients and controls are listed in Textbox 1. The age of the control group will be determined in agreement with the average age of occurrence of the CCRCC patients included in the study.

**Inclusion and exclusion criteria of participants.**

**Inclusion criteria for patients**

- Adult patient >18 years old
- All patients with a renal mass and scheduled for surgery (partial or total nephrectomy)
- Patients having accepted and signed the consent form
- Patients benefitting from social security

**Inclusion criteria for controls**

- Adult patient >18 years old
- Patients hospitalized in the urology department without a known cancer
- Patient having accepted and signed the consent form
- Patients benefitting from social security

**Exclusion criteria**

- Insufficient volume of urine sample
- Patients with a urinary catheter
- Patients under court-ordered guardianship or curatorship

### Study Objectives and Endpoints

Since CD63, CD9, and CD81 are the common markers for exosomes, we will test these three markers for detecting urinary exosomes. CA9 and vascular endothelial growth factor receptor 2 (VEGFR2) are two markers for CCRCC, which are used as markers for tumor exosomes.

The primary objective of this study is to detect urinary exosomes from CCRCC using an exosome marker (CD63) and CCRCC marker (CA9).

The secondary objectives are to assess (1) agreement between identification of CD63+/CA9+ tumor exosomes and patient status (clear cell kidney cancer), (2) agreement between the identification of CD9+/CD63+/CD81+/CA9+ tumor exosomes and patient status (clear cell kidney cancer), and (3) agreement between the identification of CD63+/VEGFR2+ tumor exosomes and patient status (clear cell kidney cancer).

The primary endpoint of the study is to characterize patients for whom CD63+ and CA9+ exosomes are detected. The secondary endpoints are as follows: (1) percentage of CD63+/CA9+ exosomes in patients with CCRCC compared with that of controls, (2) percentage of CD9+/CD63+/CD81+/CA9+ exosomes in patients with CCRCC compared with that of controls, and (3) percentage of CD63+/VGEFR2+ exosomes in patients with CCRCC compared with that of controls.

### Sample Analysis

Exosomes will be isolated using a well-established commercial kit and will be characterized using exosome markers. The tumor markers will be analyzed by transmission electronic microscopy, reverse transcription-quantitative polymerase chain reaction (RT-qPCR), and flow cytometry. Transmission electronic microscopy is typically used to characterize exosomal markers. RT-qPCR is a well-established tool for detecting gene expression and is frequently used in exosome analysis. However, conventional flow cytometry may be inconvenient for exosome analysis given the small size of exosomes. Therefore, we will use aldehyde/sulfate latex beads to capture the exosome. This technique was recently proposed to advance conventional flow cytometry as a suitable tool for analyzing exosomal markers [7].

### Data Collection

Data collected during the study will be recorded on a case report form for patients and control subjects. On the initial visit, age, sex, and medical history will be noted. For patients, date of disease diagnosis, surgical procedures, and pathological results will be noted.

### Sample Size and Statistical Analysis

A selected group of 60 patients with renal cancer and 40 control subjects will be included in the study (Figure 1). The aim of this project is to evaluate the feasibility of detecting tumor exosomes as a liquid biopsy for CCRCC. We assume a 90% assay feasibility rate, 5% unilateral α, 90% power, and 10% patient withdrawal before the initial assay. According to the one-step Fleming design, 60 patients are required. The assays of the 60 patients will be compared with those of controls to evaluate if the tumor exosomes could be cancer markers.

The diagnostic performance of tumor exosomes will be evaluated by receiver operating characteristic curves. The area under the curve will be determined with its 95% CI. Analyses will be performed using SAS 9.4.

### Patient and Public Involvement

The ethics committee examined the information notice that will be given to participants of this project. This consultation helped to improve the patient information notice on the design and aim of the study so that the participants will clearly understand and decide whether to participate in this study, in agreement with the principle of informed consent.

## Results

This study is currently underway with funding support from the CHU Saint-Etienne of France. Recruitment of all patients will be completed in 2 years. The results of the study will then be communicated via presentations and publications.

## Discussion

Research on liquid biopsy has become a hot topic. Liquid biopsy allows for the detection of tumor markers in bodily fluids for the management of cancer patients. Compared with tissue biopsy, liquid biopsy is noninvasive. Liquid biopsy usually utilizes circulating tumor cells, cell-free DNA, and exosomes. Compared with circulating tumor cells and cell-free DNA, the use of exosomes is relatively more recent, but has shown rapid development. Exosomes carry tumor markers such as nucleic acids, proteins, and lipids, including mRNAs, microRNAs, and signaling molecules that reflect the physiological and pathological condition of the cells of origin [4]. The bilayer lipid membrane of the exosome considerably improves the stability of the internal components; exosomes are therefore a rich source of biomarkers. Exosomes have recently been the subject of research into biomarkers, which hold great promise for cancers [7]. Significant advances in exosome research have been made in body fluids such as saliva, serum, urine, and amniotic fluid [5]. Blood is particularly interesting as a source of liquid biopsy since it contains cancer-specific markers in extracellular vesicles. Currently, most of the research in this field focuses on blood exosomes. Urinary exosomes are also an excellent resource for biomarkers and a promising noninvasive diagnostic instrument for kidney disease [8-11]. Moreover, urine is the easiest available bodily fluid.

Our previous study demonstrated that CA9 is a powerful marker for the diagnosis of CCRCC [12]. Exosomes from the renal tumor could be discharged into the urine since urine is in close contact with the tumor. Thus, we suggest that urinary exosomal CA9 could be used as a biomarker in the diagnosis of CCRCCs. For this purpose, we have designed a pilot study, named PEP-C, to demonstrate whether it is possible to detect exosomal CA9 in the urine of patients with CCRCC. We expect that exosomal CA9 will be detectable in the urine of patients suffering from CCRCCs when compared with that of control subjects. A cutoff corresponding to cancer detection will be determined. Moreover, we will test urinary exosomal VEGFR2 expression since VEGFR2 was found to be highly expressed in CCRCCs [13]. The exosome markers CD9, CD63, and CD81 will also be analyzed as references. These markers will be assessed using transmission electron microscopy, flow cytometry, and RT-qPCR techniques [14,15]. Our aim is to develop a liquid biopsy assay for the diagnosis of CCRCC. In the future, we hope to demonstrate that urinary exosomes could be a powerful tool to diagnose cancer and to guide clinicians in therapeutic decision-making.

**Figure 2:**
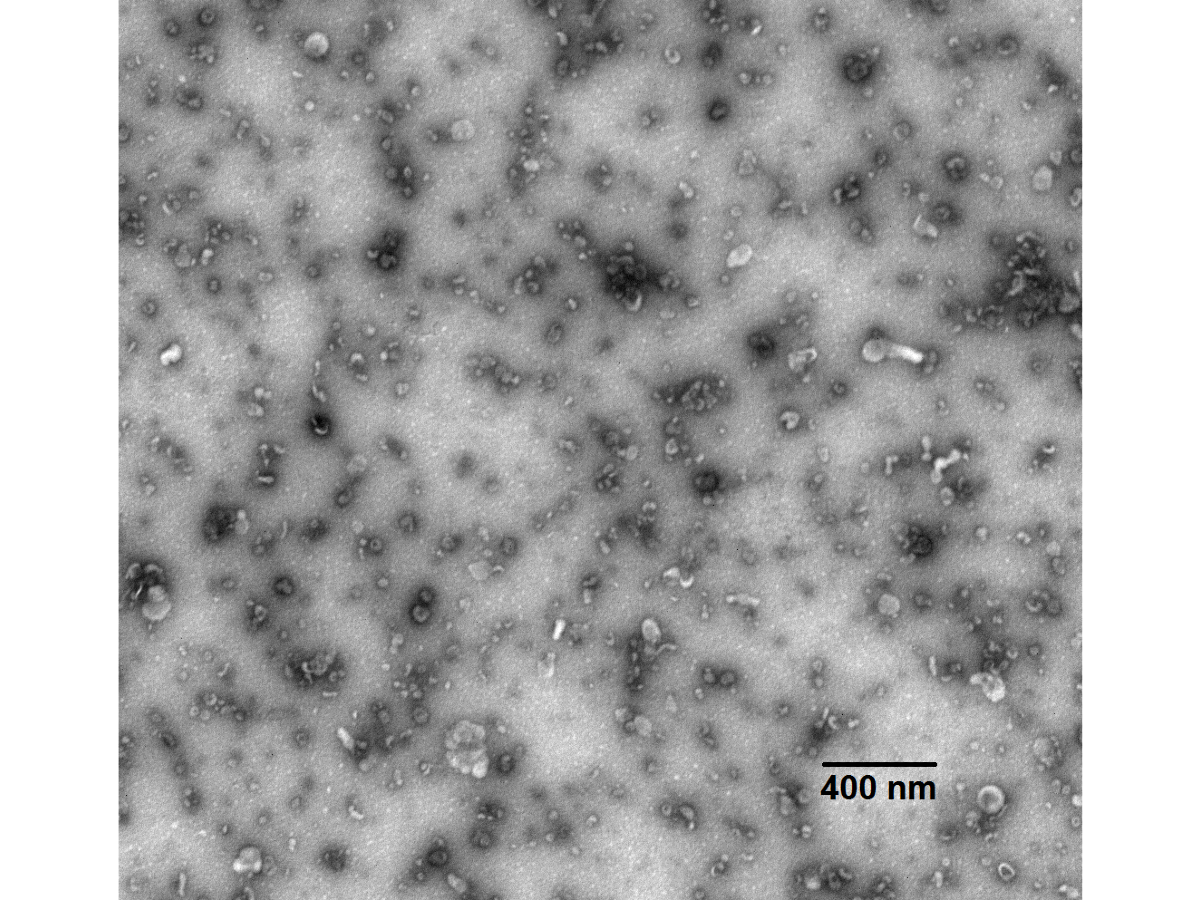
Transmission electron microscopy observation of urinary exosomes.

Several techniques for the isolation of urinary exosomes have been proposed, which can be classified according to the biochemical principle of purification: ultracentrifugation, filtration, immunocapture, or precipitation [16,17]. Ultracentrifugation isolation is the standard technique for obtaining exosomes. However, ultracentrifuges are not routinely available in clinical laboratories and this method requires a relatively long preparation time. Manufacturers have developed reagents for isolating exosomes from large volumes of urine, such as ExoQuick-TC (System Biosciences). This type of process makes clinical application possible. In preliminary tests for this project, we observed the urinary exosomes by transmission electron microscopy (Figure 2). Typical cup-shaped exosomes were observed. The size and the morphology are two important indicators for defining the urinary exosomes. Our preliminary results showed that urinary exosomes were abundant from cancer patients. Research on liquid biopsies using urine for CCRCC is very new. To our knowledge, there is almost no publication in this area. In contrast, prostate cancer–specific genes have been detected in urinary exosomes, constituting a new liquid biopsy tool to diagnose high-risk prostate cancer [18,19]. This technique of urine liquid biopsy was developed exclusively for prostate cancer and no such diagnostic method is available for CCRCC at present.

In conclusion, this will be the first study to evaluate the technical feasibility of detecting urinary exosomal CA9 in CCRCC patients. The results of this study might highlight the strong potential of liquid biopsy through urinary exosomes in the diagnosis of CCRCC. Therefore, if this pilot study is successful, a multicenter study will be envisaged.

## Appendix

Multimedia Appendix 1 External peer report A from CHU St-Etienne.

Multimedia Appendix 2 External peer report B from CHU St-Etienne.

Multimedia Appendix 3 External peer report C from CHU St-Etienne.

## Abbreviations

CCRCC: clear cell renal cell carcinoma
RT-qPCR: reverse transcription-quantitative polymerase chain reaction
VEGFR2: vascular endothelial growth factor receptor 2

## References

[1] Ljungberg B, Bensalah K, Canfield S, Dabestani S, Hofmann F, Hora M, Kuczyk MA, Lam T, Marconi L, Merseburger AS, Mulders P, Powles T, Staehler M, Volpe A, Bex A (2015). EAU guidelines on renal cell carcinoma: 2014 update. Eur Urol.

[2] Li G, Cuilleron M, Gentil-Perret A, Tostain J (2004). Characteristics of image-detected solid renal masses: implication for optimal treatment. Int J Urol.

[3] Palmirotta R, Lovero D, Cafforio P, Felici C, Mannavola F, Pellè E, Quaresmini D, Tucci M, Silvestris F (2018). Liquid biopsy of cancer: a multimodal diagnostic tool in clinical oncology. Ther Adv Med Oncol.

[4] Keller S, Ridinger J, Rupp A, Janssen JW, Altevogt P (2011). Body fluid derived exosomes as a novel template for clinical diagnostics. J Transl Med.

[5] Yang J, Wei F, Schafer C, Wong DTW (2014). Detection of tumor cell-specific mRNA and protein in exosome-like microvesicles from blood and saliva. PLoS One.

[6] Zhao A, Péoc'h M, Cottier M, Genin C, Mottet N, Li G (2015). Cell-free RNA content in urine as a possible molecular diagnostic tool for clear cell renal cell carcinoma. Int J Cancer.

[7] Melo SA, Luecke LB, Kahlert C, Fernandez AF, Gammon ST, Kaye J, LeBleu VS, Mittendorf EA, Weitz J, Rahbari N, Reissfelder C, Pilarsky C, Fraga MF, Piwnica-Worms D, Kalluri R (2015). Glypican-1 identifies cancer exosomes and detects early pancreatic cancer. Nature.

[8] Miranda KC, Bond DT, McKee M, Skog J, Păunescu TG, Da Silva N, Brown D, Russo LM (2010). Nucleic acids within urinary exosomes/microvesicles are potential biomarkers for renal disease. Kidney Int.

[9] Murakami T, Oakes M, Ogura M, Tovar V, Yamamoto C, Mitsuhashi M (2014). Development of glomerulus-, tubule-, and collecting duct-specific mRNA assay in human urinary exosomes and microvesicles. PLoS One.

[10] Spanu S, van Roeyen CRC, Denecke B, Floege J, Mühlfeld AS (2014). Urinary exosomes: a novel means to non-invasively assess changes in renal gene and protein expression. PLoS One.

[11] Street J, Koritzinsky E, Glispie D, Star R, Yuen P (2017). Urine exosomes: an emerging trove of biomarkers. Adv Clin Chem.

[12] Li G, Cuilleron M, Cottier M, Gentil-Perret A, Lambert C, Genin C, Tostain J (2006). The use of MN/CA9 gene expression in identifying malignant solid renal tumors. Eur Urol.

[13] Song SH, Jeong IG, You D, Hong JH, Hong BS, Song C, Joung JY, Moon KH, Cho YM, Ahn H, Kim C (2013). VEGF/VEGFR2 or PDGF-β/PDGFR-β expression in non-metastatic, renal cell carcinoma: a prospective study with 1,091 consecutive cases. J Urol.

[14] Jung MK, Mun JY (2018). Sample preparation and imaging of exosomes by transmission electron microscopy. J Vis Exp.

[15] Pospichalova V, Svoboda J, Dave Z, Kotrbova A, Kaiser K, Klemova D, Ilkovics L, Hampl A, Crha I, Jandakova E, Minar L, Weinberger V, Bryja V (2015). Simplified protocol for flow cytometry analysis of fluorescently labeled exosomes and microvesicles using dedicated flow cytometer. J Extracell Vesicles.

[16] Peterson MF, Otoc N, Sethi JK, Gupta A, Antes TJ (2015). Integrated systems for exosome investigation. Methods.

[17] Wu Y, Deng W, Klinke DJ (2015). Exosomes: improved methods to characterize their morphology, RNA content, and surface protein biomarkers. Analyst.

[18] McKiernan J, Donovan MJ, O'Neill V, Bentink S, Noerholm M, Belzer S, Skog J, Kattan MW, Partin A, Andriole G, Brown G, Wei JT, Thompson IM, Carroll P (2016). A novel urine exosome gene expression assay to predict high-grade prostate cancer at initial biopsy. JAMA Oncol.

[19] McKiernan J, Donovan MJ, Margolis E, Partin A, Carter B, Brown G, Torkler P, Noerholm M, Skog J, Shore N, Andriole G, Thompson I, Carroll P (2018). A prospective adaptive utility trial to validate performance of a novel urine exosome gene expression assay to predict high-grade prostate cancer in patients with prostate-specific antigen 2-10 ng/ml at initial biopsy. Eur Urol.

