# Supplementary material for: Presence of Urinary Exosomes for Liquid Biopsy of Clear Cell Renal Cell Carcinoma: Protocol for a Pilot Feasibility Study
Source: JMIR Res Protoc. 2021 Jul 20;10(7):e24423. doi: 10.2196/24423 (PMC8335600; doi:10.2196/24423)
Supplement: Multimedia Appendix 1 [file resprot_v10i7e24423_app1.pdf]

Annexe IV : Grille type expert pour projets AOL

Grille d'évaluation d'un protocole
Appel d'Offres Local 2019

DRCI CHU de ST ETIENNE

**Intitulé du projet de recherche**
**Evaluation de la présence urinaire d'exosomes provenant d'un cancer du rein à cellules claires (étude monocentrique)**
**Nom de l'Investigateur Principal**
**Pr. Nicolas MOTTET**
**Service - Etablissement de Rattachement**
**Service d'Urologie CHU St Etienne**
**Autres Centres impliqués**

**Coût total de l'étude :**<br><br>**Budget demandé dans le cadre de l'AOL ?**<br><br>**Existence d'autres sources de financement ?**<br>Si oui : Montant déjà accordé :<br>Montant en cours de demande :	**32 156 €**<br><br>**27 976 €**<br><br>**Oui |_X_|** **Non |__|**<br><br>**2674 + 1506 €**<br>**………………………….. €**
**Etude déjà débutée**	**Oui |__| ....Non |_X_|**<br><br>**Si oui, identité du promoteur :**

Explications pour l'Expert ✐

Merci de remplir la grille d'évaluation : elle comporte plusieurs pages car elle se veut une aide à votre expertise mais elle est cependant rapide à remplir.

Les chapitres de la grille d'évaluation sont les suivants **:**

- *Argumentation scientifique de l'étude*
- *Faisabilité*
- *Méthodologie*
- *Autres Informations*

Chaque chapitre comportera plusieurs items. Il s'agit de noter chaque item en inscrivant votre note en bas à droite de l'item correspondant.

- A+ = Très bon
- A = Bon
- B = Moyen
- C = Insuffisant

Votre notation doit être justifiée par un commentaire.

Si une question posée est en dehors de votre compétence d'expertise **:**

- *noter «* ***HC » = « hors compétence »*** *dans la case prévue pour la notation.*

Si le projet ne peut être concerné par la question posée :

- *cocher la case* ***« non applicable »*** *en la justifiant. Pour certains critères, cette solution n'est pas proposée car une notation est obligatoire.*

Tableau synthétique des cotations : Nous vous demandons de reporter vos notations des différents items évalués et de donner votre note finale pour l'ensemble du projet. Nous vous remercions d'utiliser la cotation suivante :

- **A^+ = Très bon projet pouvant être accepté en l'état**
- **A = Bon projet nécessitant quelques modifications mineures**
- **B = Projet moyen nécessitant des modifications majeures**
- **C = Projet non recevable en l'état**

Nous vous remercions vivement de votre collaboration.

A. ARGUMENTATION SCIENTIFIQUE

**a. Originalité de l'étude** *L'innovation proposée facilitera-t-elle une publication internationale ?*	
Commentaires :<br><br>Le projet est innovant. L'intérêt de rechercher si certains exosomes pourraient être prédictifs ou non de la présence d'un cancer du rein a cellules claires. Cette recherche de biomarqueurs est majeure dans le domaine médicale. A ma connaissance, aucun de ces exosomes n'a été étudié d'où l'originalité de l'étude. Une publication internationale est très probable si l'étude est positive	
**A+ = très bon ; A= bon ; B = moyen ; C = insuffisant ; HC = Hors compétence**	**A+**

**b. Justification de l'étude / Bibliographie** *Les données de la santé publique ou de la littérature scientifique peuvent-elles justifier l'objectif principal de l'étude ?*	
Commentaires :<br><br>Les justifications de l'étude sont clairement exposés et scientifiquement très recevables. Isoler surtout avec une valeur prédictive négative forte des biomarqueurs pourraient éviter des chirurgies rénales ou des biopsies vulnérantes inutiles.	
**A+ = très bon ; A= bon ; B = moyen ; C = insuffisant ; HC = Hors compétence**	**A**

**c. Utilité clinique de l'étude** *Les résultats issus de l'étude permettront-ils une innovation diagnostique ou thérapeutique sensible, voire une meilleure compréhension de la physiologie ou de la physiopathologie ?*	
Commentaires :<br>L'espoir est d'obtenir une très forte VPN dans cette étude. Cette étude non vulnérante peut avoir la chance de l'obtenir. Si tel est le cas, la prise en charge d'une tumeur rénale sur le plan diagnostic peut être profondément modifiée.	
**A+ = très bon ; A= bon ; B = moyen ; C = insuffisant ; HC = Hors compétence**	**A+**

B. FAISABILITE

**a. Potentiel de recrutement** *L'activité des centres investigateurs, la prévalence de la maladie étudiée, la taille et la disponibilité de la population souche permettront-elles la réalisation des inclusions voulues dans un temps raisonnable ?Existe-t-il à votre connaissance des études concurrentielles ?*
Commentaires :<br><br>Le centre d'urologie de saint Etienne a calculé le nombre de patients nécessaires en fonction du nombre de chirurgie pour ce diagnostic fait annuellement. Ainsi les nombres avancés sont factuels et donc sans problème de recrutement. De plus, ne demandant qu'une analyse d'urine , il parait certain que le patient accepte ce type d'étude. Aucun travail concurrentiel en recherchant sur clinical trial

**A+ = très bon ; A= bon ; B = moyen ; C = insuffisant ; HC = Hors compétence**	**A+**
Non applicable ☐ Justifier :	

**b. Organisation pratique** *La logistique proposée permettra-t-elle l'inclusion et le suivi des patients, ainsi que l'analyse des données ?*
Commentaires :<br><br>Clairement décrite par les auteurs. Aucun commentaire particulier

**A+ = très bon ; A= bon ; B = moyen ; C = insuffisant ; HC = Hors compétence**	**A**

**c. Considérations éthiques / Protection des personnes**
Commentaires :<br><br>Pas de commentaires car étude sans bénéfice directe annoncée, sans risque et avec une lettre au patient claire pour une analyse urinaire non vulnérante

**A+ = très bon ; A= bon ; B = moyen ; C = insuffisant ; HC = Hors compétence**	**A+**

**d. Adéquation des moyens humains, matériels et financiers avec l'étude** *Un contrôle de qualité est-il prévu et adapté ? Y a-t-il du personnel spécifique (TEC, ARC…) pour la réalisation de l'étude ? Un comité de surveillance et un comité de validation des événements indésirables grave sont-ils prévus ? La somme demandée est-elle adaptée à la réalisation du projet ?*
Commentaires :<br>Les moyens humains pour ce travail sont en adéquation avec l'étude. Seul point a éclaircir, qui fera l'analyse statistique. Probablement les auteurs eux même car aucun surcout n'est prévu

**A+ = très bon ; A= bon ; B = moyen ; C = insuffisant; HC = Hors compétence**	**A**

C. METHODOLOGIE

**a. Objectifs / Critères d'évaluation** *Y a-t-il définition d'un objectif principal pertinent en regard de la problématique ? Y a-t-il adéquation entre les objectifs et les critères d'évaluation ?*	
Commentaires :<br>Les objectifs ainsi que les critères d'évaluation sont clairement établis	
**A+ = très bon ; A= bon ; B = moyen ; C = insuffisant ; HC = Hors compétence**	**A**

**b. Plan expérimental** *Est-il adapté pour répondre à l'objectif principal ? En cas d'étude randomisée, la procédure de randomisation est-elle détaillée et adaptée ?*	
Commentaires :<br>Seul point qui sera important pour l'avenir. Il serait intéressant d'avoir des données sur la variabilité intra et interindividuel du test. A rapporter pour une publication	
**A+ = très bon ; A= bon ; B = moyen ; C = insuffisant ; HC = Hors compétence**	**A-**
Non applicable ☐ Justifier :	

**c. Population étudiée** *Les critères d'inclusion et de non- inclusion proposés sont-ils pertinents ?*	
Commentaires :<br>Les deux groupes sont clairement décrits par les auteurs ; pas de commentaires	
**A+ = très bon ; A= bon ; B = moyen ; C = insuffisant ; HC = Hors compétence**	**A+**
Non applicable ☐ Justifier :	

**d. Calcul du nombre de sujets / Analyse statistique** *La méthode utilisée pour le calcul est-elle pertinente ? Les tests proposés sont-ils adaptés ?*	
Commentaires :<br>N'ayant aucune donnée sur un travail original et exploratoire ; les nombres choisis sont ceux possibles dans la réalité. Cette hypothèse est donc acceptable.	
**A+ = très bon ; A= bon ; B = moyen ; C = insuffisant ; HC = Hors compétence**	**A**
Non applicable ☐ Justifier :	

D. AUTRES INFORMATIONS

**a. Déroulement / Règles d'arrêt**<br>*Le déroulement est-il suffisamment précis ? En cas d'étude nécessitant, à votre avis, un comité de surveillance, celui-ci est-il prévu ? Y a-t-il définition des règles d'arrêt de l'étude ?*	
Commentaires :<br><br>Clairement identifiés. Pas d'arrêt de principe car analyse uniquement urinaire	
**A+ = très bon ; A= bon ; B = moyen ; C = insuffisant ; HC = Hors compétence**	A+

**b. Demande financière** *La demande est-elle adaptée / appel d'offres et justifiée / projet ?*	
Commentaires :<br>La grille budgétaire est claire et en corrélation avec l'étude. Un don complémentaire a déjà été obtenu.	
**A+ = très bon ; A= bon ; B = moyen ; C = insuffisant ; HC = Hors compétence**	A+

**c. Qualité rédactionnelle et présentation** *(respect du plan-type ANSM / DGS ?)*	
Tres clair	
**A+ = très bon ; A= bon ; B = moyen ; C = insuffisant ; HC = Hors compétence**	A

Commentaires libres / Conclusions

1. **D'ordre scientifique**

La recherche du Graal est toujours un but en médecine. Les auteurs peuvent avoir la chance de le trouver. En tout cas, on l'espère pour eux et les patients

2. **D'ordre financier**

Pas de commentaires

Rapport synthétique

Merci de reporter ici vos notes par item

Le signe « / » veut dire que vous ne pouvez pas cocher « NA » car la cotation de ce critère est obligatoire.

CRITERES DE LA GRILLE D'EVALUATION	A^+	A	B	C	HC	NA
**A. Argumentation scientifique**						
a. Originalité de l'étude	**A+**					/
b. Justification de l'étude / Bibliographie		**A**				
c. Utilité clinique de l'étude	**A+**					/
**B. Faisabilité**						
a. Potentiel de recrutement	**A+**					
b. Organisation pratique		**A**				
c. Considérations éthiques et protection des personnes	**A+**					/
d. Adéquation des moyens humains, matériels et financiers		**A**				/
**C. Méthodologie**						
a. Objectifs / Critères d'évaluation	**A+**					/
b. Plan expérimental		**A**				
c. Population étudiée	**A+**					
d. Calcul du nombre de sujets / Analyse statistique		**A**				
**D. Informations générales**						
a. Déroulement / Règles d'arrêt	**A+**					
b. Demande financière adaptée ?	**A+**					/
c. Qualité rédactionnelle / Présentation		**A**				/

**Note globale donnée au projet (A^+, A, B ou C)**	**A+**

- **A^+ = Très bon projet pouvant être accepté en l'état**
- **A = Bon projet nécessitant quelques modifications mineures**
- **B = Projet moyen nécessitant des modifications majeures**
- **C = Projet non recevable en l'état**

La note globale doit tenir compte de la pondération éventuelle que vous souhaitez apporter à certains des critères selon l'importance que vous leur attribuez.

Compte-tenu des résultats des années précédentes, une note B globale peut compromettre l'acceptation du projet.
