# Supplementary material for: Presence of Urinary Exosomes for Liquid Biopsy of Clear Cell Renal Cell Carcinoma: Protocol for a Pilot Feasibility Study
Source: JMIR Res Protoc. 2021 Jul 20;10(7):e24423. doi: 10.2196/24423 (PMC8335600; doi:10.2196/24423)
Supplement: Multimedia Appendix 2 [file resprot_v10i7e24423_app2.pdf]

Annexe IV : Grille type expert pour projets AOL

**Grille d'évaluation d'un protocole**<br>**Appel d'Offres Local 2019**<br><br>**DRCI CHU de ST ETIENNE**

**Intitulé du projet de recherche**
**Evaluation de la présence urinaire d'exosomes provenant d'un cancer du rein à cellules claires (étude monocentrique)**
**Nom de l'Investigateur Principal**
**Pr. Nicolas MOTTET**
**Service - Etablissement de Rattachement**
**Service d'Urologie CHU St Etienne**
**Autres Centres impliqués**

**Coût total de l'étude :**<br><br>**Budget demandé dans le cadre de l'AOL ?**<br><br>**Existence d'autres sources de financement ?**<br>Si oui : Montant déjà accordé :<br>Montant en cours de demande :	**32 156 €**<br><br>**27 976 €**<br><br>**Oui |_X_|** **Non |__|**<br><br>**2674 + 1506 €**<br>**………………………….. €**
**Etude déjà débutée**	**Oui |__|** **..................Non |_X_|**<br><br>**Si oui, identité du promoteur :**

Explications pour l'Expert ✐

Merci de remplir la grille d'évaluation : elle comporte plusieurs pages car elle se veut une aide à votre expertise mais elle est cependant rapide à remplir.

Les chapitres de la grille d'évaluation sont les suivants :

- *Argumentation scientifique de l'étude*
- *Faisabilité*
- *Méthodologie*
- *Autres Informations*

Chaque chapitre comportera plusieurs items. Il s'agit de noter chaque item en inscrivant votre note en bas à droite de l'item correspondant.

- A+ = Très bon
- A = Bon
- B = Moyen
- C = Insuffisant

Votre notation doit être justifiée par un commentaire.

Si une question posée est en dehors de votre compétence d'expertise :

- *noter « **HC** » = « **hors compétence** » dans la case prévue pour la notation.*

Si le projet ne peut être concerné par la question posée :

- *cocher la case « **non applicable** » en la justifiant. Pour certains critères, cette solution n'est pas proposée car une notation est obligatoire.*

Tableau synthétique des cotations : Nous vous demandons de reporter vos notations des différents items évalués et de donner votre note finale pour l'ensemble du projet. Nous vous remercions d'utiliser la cotation suivante :

- **A^+ = Très bon projet pouvant être accepté en l'état**
- **A = Bon projet nécessitant quelques modifications mineures**
- **B = Projet moyen nécessitant des modifications majeures**
- **C = Projet non recevable en l'état**

Nous vous remercions vivement de votre collaboration.

A. ARGUMENTATION SCIENTIFIQUE

**a. Originalité de l'étude** *L'innovation proposée facilitera-t-elle une publication internationale ?*	
Commentaires :<br>Étude innovante dans le domaine du cancer rénal. Il n'y a pas de données validées à ce sujet dans la littérature à ce jour.<br>Le sujet est donc pertinent et vierge. Le potentiel de publication est donc très important.	
**A+ = très bon ; A= bon ; B = moyen ; C = insuffisant ; HC = Hors compétence**	**A+**

**b. Justification de l'étude / Bibliographie** *Les données de la santé publique ou de la littérature scientifique peuvent-elles justifier l'objectif principal de l'étude ?*	
Commentaires :<br>Cf. paragraphe précédent. L'absence de données à ce sujet justifie en soit l'étude.<br>En revanche l'implication clinique des résultats attendus aurait pu être plus détaillée.	
**A+ = très bon ; A= bon ; B = moyen ; C = insuffisant ; HC = Hors compétence**	**A**

**c. Utilité clinique de l'étude** *Les résultats issus de l'étude permettront-ils une innovation diagnostique ou thérapeutique sensible, voire une meilleure compréhension de la physiologie ou de la physiopathologie ?*	
Commentaires :<br>Oui, ces résultats peuvent avoir une utilité clinique à court terme. Ce point aurait mérité d'être plus détaillé.	
**A+ = très bon ; A= bon ; B = moyen ; C = insuffisant ; HC = Hors compétence**	**A**

B. FAISABILITE

**a. Potentiel de recrutement** *L'activité des centres investigateurs, la prévalence de la maladie étudiée, la taille et la disponibilité de la population souche permettront-elles la réalisation des inclusions voulues dans un temps raisonnable ?Existe-t-il à votre connaissance des études concurrentielles ?*	
Commentaires :<br>Ce point est bien détaillé et semble parfaitement anticipé avant un plan de recrutement raisonnable.<br>Il n'y a pas d'étude concurrente potentielle.	
**A+ = très bon ; A= bon ; B = moyen ; C = insuffisant ; HC = Hors compétence**	A+
Non applicable ☐ Justifier :	

**b. Organisation pratique** *La logistique proposée permettra-t-elle l'inclusion et le suivi des patients, ainsi que l'analyse des données ?*	
Commentaires :<br>Ce point ne devrait pas non plus poser de problème. Inclusion et suivi simples à mettre en œuvre.	
**A+ = très bon ; A= bon ; B = moyen ; C = insuffisant ; HC = Hors compétence**	A+

**c. Considérations éthiques / Protection des personnes**	
Commentaires : Ce point a été bien pris en compte et détaillé. Pas de commentaire	
**A+ = très bon ; A= bon ; B = moyen ; C = insuffisant ; HC = Hors compétence**	A+

**d. Adéquation des moyens humains, matériels et financiers avec l'étude** *Un contrôle de qualité est-il prévu et adapté ? Y a-t-il du personnel spécifique (TEC, ARC…) pour la réalisation de l'étude ? Un comité de surveillance et un comité de validation des événements indésirables grave sont-ils prévus ? La somme demandée est-elle adaptée à la réalisation du projet ?*	
Commentaires :<br>Le détail du prévisionnel des moyens nécessaires semble cohérent en regard du sujet.	
**A+ = très bon ; A= bon ; B = moyen ; C = insuffisant; HC = Hors compétence**	A

C. METHODOLOGIE

**a. Objectifs / Critères d'évaluation** *Y a-t-il définition d'un objectif principal pertinent en regard de la problématique ? Y a-t-il adéquation entre les objectifs et les critères d'évaluation ?*	
Commentaires : Les objectifs et critères de jugement sont correctement décrits et pertinents aux vues du sujet	
**A+ = très bon ; A= bon ; B = moyen ; C = insuffisant ; HC = Hors compétence**	**A**

**b. Plan expérimental** *Est-il adapté pour répondre à l'objectif principal ? En cas d'étude randomisée, la procédure de randomisation est-elle détaillée et adaptée ?*	
Commentaires :<br>Le plan est adapté. Pas de commentaire	
**A+ = très bon ; A= bon ; B = moyen ; C = insuffisant ; HC = Hors compétence**	**A+**
Non applicable ☐ Justifier :	

**c. Population étudiée** *Les critères d'inclusion et de non- inclusion proposés sont-ils pertinents ?*	
Commentaires : Ces critères sont pertinents	
**A+ = très bon ; A= bon ; B = moyen ; C = insuffisant ; HC = Hors compétence**	**A**
Non applicable ☐ Justifier :	

**d. Calcul du nombre de sujets / Analyse statistique** *La méthode utilisée pour le calcul est-elle pertinente ? Les tests proposés sont-ils adaptés ?*	
Commentaires : Pas de calcul d'effectif. Cela est bien justifié dans le texte du fait de l'absence de données suffisantes dans la littérature pour effectuer ce calcul.	
**A+ = très bon ; A= bon ; B = moyen ; C = insuffisant ; HC = Hors compétence**	**B**
Non applicable ☐ Justifier :	

D. AUTRES INFORMATIONS

**a. Déroulement / Règles d'arrêt**<br>*Le déroulement est-il suffisamment précis ? En cas d'étude nécessitant, à votre avis, un comité de surveillance, celui-ci est-il prévu ? Y a-t-il définition des règles d'arrêt de l'étude ?*	
Commentaires : Non applicable	
**A+ = très bon ; A= bon ; B = moyen ; C = insuffisant ; HC = Hors compétence**	**A**

**b. Demande financière** *La demande est-elle adaptée / appel d'offres et justifiée / projet ?*	
Commentaires :<br>Selon le budget prévisionnel fourni, la demande semble justifiée.	
**A+ = très bon ; A= bon ; B = moyen ; C = insuffisant ; HC = Hors compétence**	**A**

**c. Qualité rédactionnelle et présentation** *(respect du plan-type ANSM / DGS ?)*	
Projet bien rédigé, plan cohérent, agréable à lire.<br>Les informations sont simples à retrouver dans le document.<br>Résumé clair<br>Flow chart très synthétique et informatif	
**A+ = très bon ; A= bon ; B = moyen ; C = insuffisant ; HC = Hors compétence**	**A**

**Commentaires libres / Conclusions**
**1. D'ordre scientifique**<br>**Étude pertinente pouvant aboutir à des résultats ayant un impact sur la pratique clinique à court terme.**<br><br>**2. D'ordre financier**<br>Pas de commentaire.

Rapport synthétique

Merci de reporter ici vos notes par item

Le signe « / » veut dire que vous ne pouvez pas cocher « NA » car la cotation de ce critère est obligatoire.

CRITERES DE LA GRILLE D'EVALUATION	A^+	A	B	C	HC	NA
**A. Argumentation scientifique**						
a. Originalité de l'étude	X					/
b. Justification de l'étude / Bibliographie		X				
c. Utilité clinique de l'étude		X				/
**B. Faisabilité**						
a. Potentiel de recrutement	X					
b. Organisation pratique	X					
c. Considérations éthiques et protection des personnes	X					/
d. Adéquation des moyens humains, matériels et financiers		X				/
**C. Méthodologie**						
a. Objectifs / Critères d'évaluation		X				/
b. Plan expérimental	X					
c. Population étudiée		X				
d. Calcul du nombre de sujets / Analyse statistique			X			
**D. Informations générales**						
a. Déroulement / Règles d'arrêt		X				
b. Demande financière adaptée ?		X				/
c. Qualité rédactionnelle / Présentation		X				/

**Note globale donnée au projet (A^+, A, B ou C)**	**A**

- **A^+ = Très bon projet pouvant être accepté en l'état**
- **A = Bon projet nécessitant quelques modifications mineures**
- **B = Projet moyen nécessitant des modifications majeures**
- **C = Projet non recevable en l'état**

La note globale doit tenir compte de la pondération éventuelle que vous souhaitez apporter à certains des critères selon l'importance que vous leur attribuez.

Compte-tenu des résultats des années précédentes, une note B globale peut compromettre l'acceptation du projet.
