# Supplementary material for: Presence of Urinary Exosomes for Liquid Biopsy of Clear Cell Renal Cell Carcinoma: Protocol for a Pilot Feasibility Study
Source: JMIR Res Protoc. 2021 Jul 20;10(7):e24423. doi: 10.2196/24423 (PMC8335600; doi:10.2196/24423)
Supplement: Multimedia Appendix 3 [file resprot_v10i7e24423_app3.pdf]

Annexe IV : Grille type expert pour projets AOL

Grille d'évaluation d'un protocole<br>Appel d'Offres Local 2019<br><br>DRCI CHU de ST ETIENNE

**Intitulé du projet de recherche**
**Evaluation de la présence urinaire d'exosomes provenant d'un cancer du rein à cellules claires (étude monocentrique)**
**Nom de l'Investigateur Principal**
**Pr. Nicolas MOTTET**
**Service - Etablissement de Rattachement**
**Service d'Urologie CHU St Etienne**
**Autres Centres impliqués**

**Coût total de l'étude :**<br><br>**Budget demandé dans le cadre de l'AOL ?**<br><br>**Existence d'autres sources de financement ?**<br>Si oui : Montant déjà accordé :<br>Montant en cours de demande :	**32 156 €**<br><br>**27 976 €**<br><br>**Oui |_X_| Non |__|**<br><br>**2674 + 1506 €**<br>**………………………….. €**
**Etude déjà débutée**	**Oui |__| ....Non |_X_|**<br><br>**Si oui, identité du promoteur :**

Explications pour l'Expert ✐

Merci de remplir la grille d'évaluation : elle comporte plusieurs pages car elle se veut une aide à votre expertise mais elle est cependant rapide à remplir.

Les chapitres de la grille d'évaluation sont les suivants :

- *Argumentation scientifique de l'étude*
- *Faisabilité*
- *Méthodologie*
- *Autres Informations*

Chaque chapitre comportera plusieurs items. Il s'agit de noter chaque item en inscrivant votre note en bas à droite de l'item correspondant.

- A+ = Très bon
- A = Bon
- B = Moyen
- C = Insuffisant

Votre notation doit être justifiée par un commentaire.

Si une question posée est en dehors de votre compétence d'expertise :

- *noter « **HC** » = « **hors compétence** » dans la case prévue pour la notation.*

Si le projet ne peut être concerné par la question posée :

- *cocher la case « **non applicable** » en la justifiant. Pour certains critères, cette solution n'est pas proposée car une notation est obligatoire.*

Tableau synthétique des cotations : Nous vous demandons de reporter vos notations des différents items évalués et de donner votre note finale pour l'ensemble du projet. Nous vous remercions d'utiliser la cotation suivante :

- **A^+ = Très bon projet pouvant être accepté en l'état**
- **A = Bon projet nécessitant quelques modifications mineures**
- **B = Projet moyen nécessitant des modifications majeures**
- **C = Projet non recevable en l'état**

Nous vous remercions vivement de votre collaboration.

A. ARGUMENTATION SCIENTIFIQUE

**a. Originalité de l'étude** *L'innovation proposée facilitera-t-elle une publication internationale ?*	
Commentaires :<br>La démonstration de la validité clinique et de l'utilité clinique d'exosomes (ici urinaires) en tant que biomarqueurs de la pathologie tumorale rénale (carcinome à cellules claires) afin de disposer d'une alternative (biopsie liquide) à la biopsie tissulaire est un concept intéressant, relativement peu investigué dans le cancer du rein par rapport à d'autres types tumoraux (poumon..).<br>De fait, relative bonne originalité du projet.<br>Note A	
**A+ = très bon ; A= bon ; B = moyen ; C = insuffisant ; HC = Hors compétence**	**A**

**b. Justification de l'étude / Bibliographie** *Les données de la santé publique ou de la littérature scientifique peuvent-elles justifier l'objectif principal de l'étude ?*	
Commentaires :<br>Les données bibliographiques et l'état des connaissances scientifiques actuelles justifient l'intérêt de l'étude, premier pas descriptif vers une démonstration de validité et d'utilité clinique.<br>Note A.	
**A+ = très bon ; A= bon ; B = moyen ; C = insuffisant ; HC = Hors compétence**	**A**

**c. Utilité clinique de l'étude** *Les résultats issus de l'étude permettront-ils une innovation diagnostique ou thérapeutique sensible, voire une meilleure compréhension de la physiologie ou de la physiopathologie ?*	
Commentaires :<br>Les résultats de l'étude, purement descriptif, permettront simplement de valider une première étape, celle la validité analytique de la recherche d'exosomes urinaires de tumeurs rénales grâce aux marqueurs choisis (sensibilité et la spécificité analytiques, la reproductibilité, robustesse de la méthode détection).<br>Ils ne permettront pas d'affirmer la validité clinique (sensibilité, spécificité clinique…) ou son utilité clinique (impact – diagnostic et thérapeutique - sur la prise en charge d'une tumeur rénale).<br>Note C.	
**A+ = très bon ; A= bon ; B = moyen ; C = insuffisant ; HC = Hors compétence**	**C**

B. FAISABILITE

**a. Potentiel de recrutement** *L'activité des centres investigateurs, la prévalence de la maladie étudiée, la taille et la disponibilité de la population souche permettront-elles la réalisation des inclusions voulues dans un temps raisonnable ?Existe-t-il à votre connaissance des études concurrentielles ?*
Commentaires :<br>Etude monocentrique se proposant de recruter 60 patients et 40 témoins sur 2 ans, objectif de recrutement relativement crédible et raisonnable.<br>Note A.

**A+ = très bon ; A= bon ; B = moyen ; C = insuffisant ; HC = Hors compétence**	**A**
Non applicable ☐ Justifier :	

**b. Organisation pratique** *La logistique proposée permettra-t-elle l'inclusion et le suivi des patients, ainsi que l'analyse des données ?*
Commentaires :<br>Logistique adéquate au vu de l'environnement décrit.<br>Note A.

**A+ = très bon ; A= bon ; B = moyen ; C = insuffisant ; HC = Hors compétence**	**A**

**c. Considérations éthiques / Protection des personnes**
Commentaires :<br>Cadre réglementaire adapté, document de consentement fourni, RAS.

**A+ = très bon ; A= bon ; B = moyen ; C = insuffisant ; HC = Hors compétence**	**A**

**d. Adéquation des moyens humains, matériels et financiers avec l'étude** *Un contrôle de qualité est-il prévu et adapté ? Y a-t-il du personnel spécifique (TEC, ARC…) pour la réalisation de l'étude ? Un comité de surveillance et un comité de validation des événements indésirables grave sont-ils prévus ? La somme demandée est-elle adaptée à la réalisation du projet ?*
Commentaires :<br>Budget de 28 K€ demandé à l'AO sur un total de 32 K€, adapté aux objectifs et à l'ambition de l'étude (postes majeurs de dépenses : kits de réactifs et biopathologie).<br>Note A.

**A+ = très bon ; A= bon ; B = moyen ; C = insuffisant; HC = Hors compétence**	

C. METHODOLOGIE

**a. Objectifs / Critères d'évaluation** *Y a-t-il définition d'un objectif principal pertinent en regard de la problématique ? Y a-t-il adéquation entre les objectifs et les critères d'évaluation ?*	
Commentaires :<br>L'étude a un objectif très simple, voire limité, purement descriptif, qui limité de fait l'envergure de l'étude (validité analytique), dans le cadre il est vrai d'une étude de faisabilité.<br>Note B.	
**A+ = très bon ; A= bon ; B = moyen ; C = insuffisant ; HC = Hors compétence**	**B**

**b. Plan expérimental** *Est-il adapté pour répondre à l'objectif principal ? En cas d'étude randomisée, la procédure de randomisation est-elle détaillée et adaptée ?*	
Commentaires :<br>Plan expérimental, notamment dans le cadre de la compara raison des capacités des marqueurs utilisés à détecter des exosomes spécifiques d'une tumeur rénale, réduit à sa plus simple expression.<br>Note C.	
**A+ = très bon ; A= bon ; B = moyen ; C = insuffisant ; HC = Hors compétence**	**C**
<u>Non applicable</u> ☐ Justifier :	

**c. Population étudiée** *Les critères d'inclusion et de non- inclusion proposés sont-ils pertinents ?*	
Commentaires :<br>Population éligible en adéquation avec les objectifs.<br>Note A.	
**A+ = très bon ; A= bon ; B = moyen ; C = insuffisant ; HC = Hors compétence**	**A**
<u>Non applicable</u> ☐ Justifier :	

**d. Calcul du nombre de sujets / Analyse statistique** *La méthode utilisée pour le calcul est-elle pertinente ? Les tests proposés sont-ils adaptés ?*	
Commentaires :<br>Absence complète de méthodologie (évaluation des performances du test chez les cas par rapport aux témoins ?), le protocole se limitant à quelques lignes d'analyse descriptive.<br>Note C.	
**A+ = très bon ; A= bon ; B = moyen ; C = insuffisant ; HC = Hors compétence**	**C**
<u>Non applicable</u> ☐ Justifier :	

D. AUTRES INFORMATIONS

**a. Déroulement / Règles d'arrêt**<br>*Le déroulement est-il suffisamment précis ? En cas d'étude nécessitant, à votre avis, un comité de surveillance, celui-ci est-il prévu ? Y a-t-il définition des règles d'arrêt de l'étude ?*	
Commentaires :<br>Déroulement bien explicité.<br>Note A.	
**A+ = très bon ; A= bon ; B = moyen ; C = insuffisant ; HC = Hors compétence**	**A**

**b. Demande financière** *La demande est-elle adaptée / appel d'offres et justifiée / projet ?*	
Commentaires :<br>Demande en adéquation avec les objectifs.<br>Note A.	
**A+ = très bon ; A= bon ; B = moyen ; C = insuffisant ; HC = Hors compétence**	**A**

**c. Qualité rédactionnelle et présentation** *(respect du plan-type ANSM / DGS ?)*	
Correcte, rédaction assez minimaliste.<br>Note B.	
**A+ = très bon ; A= bon ; B = moyen ; C = insuffisant ; HC = Hors compétence**	**B**

Commentaires libres / Conclusions

1. **D'ordre scientifique**

Sujet intéressant, avec une première étape descriptive d'investigation, mais cette étude de faisabilité ne documente en aucune façon la validité ni l'utilité clinique des marqueurs utilisés pour la détection d'exosomes urinaires chez les patients atteints de tumeurs rénales.
Projet très pauvre sur un plan méthodologique.

2. **D'ordre financier**

Pas de difficulté d'ordre financière, budget en adéquation avec les objectifs et l'ambition de l'étude.

Rapport synthétique

Merci de reporter ici vos notes par item

Le signe « / » veut dire que vous ne pouvez pas cocher « NA » car la cotation de ce critère est obligatoire.

CRITERES DE LA GRILLE D'EVALUATION	A+	A	B	C	HC	NA
**A. Argumentation scientifique**						
a. Originalité de l'étude		**A**				/
b. Justification de l'étude / Bibliographie		**A**				
c. Utilité clinique de l'étude				**C**		/
**B. Faisabilité**						
a. Potentiel de recrutement		**A**				
b. Organisation pratique		**A**				
c. Considérations éthiques et protection des personnes		**A**				/
d. Adéquation des moyens humains, matériels et financiers		**A**				/
**C. Méthodologie**						
a. Objectifs / Critères d'évaluation			**B**			/
b. Plan expérimental				**C**		
c. Population étudiée		**A**				
d. Calcul du nombre de sujets / Analyse statistique				**C**		
**D. Informations générales**						
a. Déroulement / Règles d'arrêt		**A**				
b. Demande financière adaptée ?		**A**				/
c. Qualité rédactionnelle / Présentation			**B**			/

**Note globale donnée au projet (A+, A, B ou C)**	**B**

- **A+ = Très bon projet pouvant être accepté en l'état**
- **A = Bon projet nécessitant quelques modifications mineures**
- **B = Projet moyen nécessitant des modifications majeures**
- **C = Projet non recevable en l'état**

La note globale doit tenir compte de la pondération éventuelle que vous souhaitez apporter à certains des critères selon l'importance que vous leur attribuez.

Compte-tenu des résultats des années précédentes, une note B globale peut compromettre l'acceptation du projet.
